# Dietary selenium intake and asthma in US children and adolescents

**DOI:** 10.1016/j.jped.2026.101551

**Published:** 2026-05-06

**Authors:** Lingyu Li, Shuai Wang, Hongyan Wei, Changcheng Sun

**Affiliations:** People's Hospital of Cangzhou, Cangzhou, China

**Keywords:** Selenium, Asthma, Child, Adolescent, Cross-sectional study

## Abstract

**Objective:**

Evidence concerning total dietary selenium intake in the US pediatric population remains sparse and inconsistent. This study employs a nationally representative dataset to investigate the association between selenium intake and asthma prevalence, addressing this critical knowledge gap.

**Method:**

The National Health and Nutrition Examination Survey (NHANES) (2011–2020) furnished data on asthmatic individuals aged < 20 years with quantified dietary selenium intake. Utilizing rigorous statistical methodologies — multivariable logistic regression, restricted cubic spline (RCS) modeling, and subgroup analyses — the authors evaluated the selenium-asthma association.

**Results:**

In this study encompassing 7780 pediatric participants, multivariable logistic regression with comprehensive covariate adjustment revealed progressively higher asthma risk with increasing selenium exposure. Compared to the lowest intake tertile (Q1: ≤ 73.3 mg/day), adjusted ORs were 1.15 (95% CI: 0.98–1.35; *p* = 0.082) for Q2 (73.4–112.6 mg/day) and 1.24 (95% CI: 1.00–1.54; *p* = 0.048) for Q3 (≥ 112.7 mg/day). Subsequent restricted cubic spline modeling quantitatively established a significant dose-response relationship (*p* = 0.042) between dietary selenium levels and asthma risk.

**Conclusion:**

Dietary selenium intake demonstrated a significant dose-dependent association with asthma prevalence among US pediatric populations.

## Introduction

Asthma remains a predominant chronic respiratory disorder in pediatric populations globally, affecting over 5.4 million children in the United States alone and imposing substantial healthcare burdens [1]. Characterized by recurrent wheezing, airflow limitation, and bronchial hyperresponsiveness, its pathogenesis involves complex gene-environment interactions where oxidative stress plays a pivotal mechanistic role [[2], [3]].

Selenium, an essential trace element integral to selenoproteins such as glutathione peroxidase, serves as a pivotal redox regulator within antioxidant defense systems [4]. Epidemiological investigations have increasingly examined its potential role in asthma pathophysiology. Contemporary research indicates U-shaped associations: both deficiency and excess selenium intake may exert adverse effects. Hoffman et al. found that both moderately selenium-deficient diets and high-selenium regimens attenuate asthma severity, whereas adequate selenium exposure paradoxically elevates asthma risk [[5], [6]]. The New Zealand pediatric cohort study demonstrated no significant epidemiological association between selenium status and elevated asthma risk [7]. Mechanistically, selenium exerts protective effects by attenuating reactive oxygen species (ROS) generation and suppressing chronic inflammation, potentially through facilitating Th1/Th2 immune balance modulation via enhanced T-helper cell responsiveness to allergens [[8], [9]]. Shaheen et al. [10]. report that suboptimal maternal serum Se levels compromise GPx function, impairing antioxidant defenses against oxidative stress within the fetal airway epithelium. This results in epithelial damage and contributes to asthma development.

Despite these advances, defining optimal selenium thresholds for pediatric asthma prevention remains elusive, with a notable scarcity of randomized controlled trials in this population. Current epidemiological evidence exhibits substantial methodological heterogeneity: multiple longitudinal cohorts report conflicting selenium-asthma associations, primarily attributable to inconsistent exposure assessment and inadequate statistical power [11]. The nationally representative NHANES survey (2011–2020) uniquely addresses these limitations through its rigorous 24-hour dietary recall methodology and capacity for comprehensive adjustment of nutritional, environmental, and demographic confounders.

The present study examined the dose-response relationship between dietary selenium intake and asthma prevalence in U.S. children and adolescents. Using multivariable logistic regression incorporating restricted cubic splines, the authors specifically assessed whether selenium exposure affects asthma risk, adjusting for nutritional and environmental covariates. These findings aim to contribute to establishing evidence-based nutritional guidelines for asthma prevention in pediatric populations.

## Method

### Data sources and study population

**Fig. 1:**
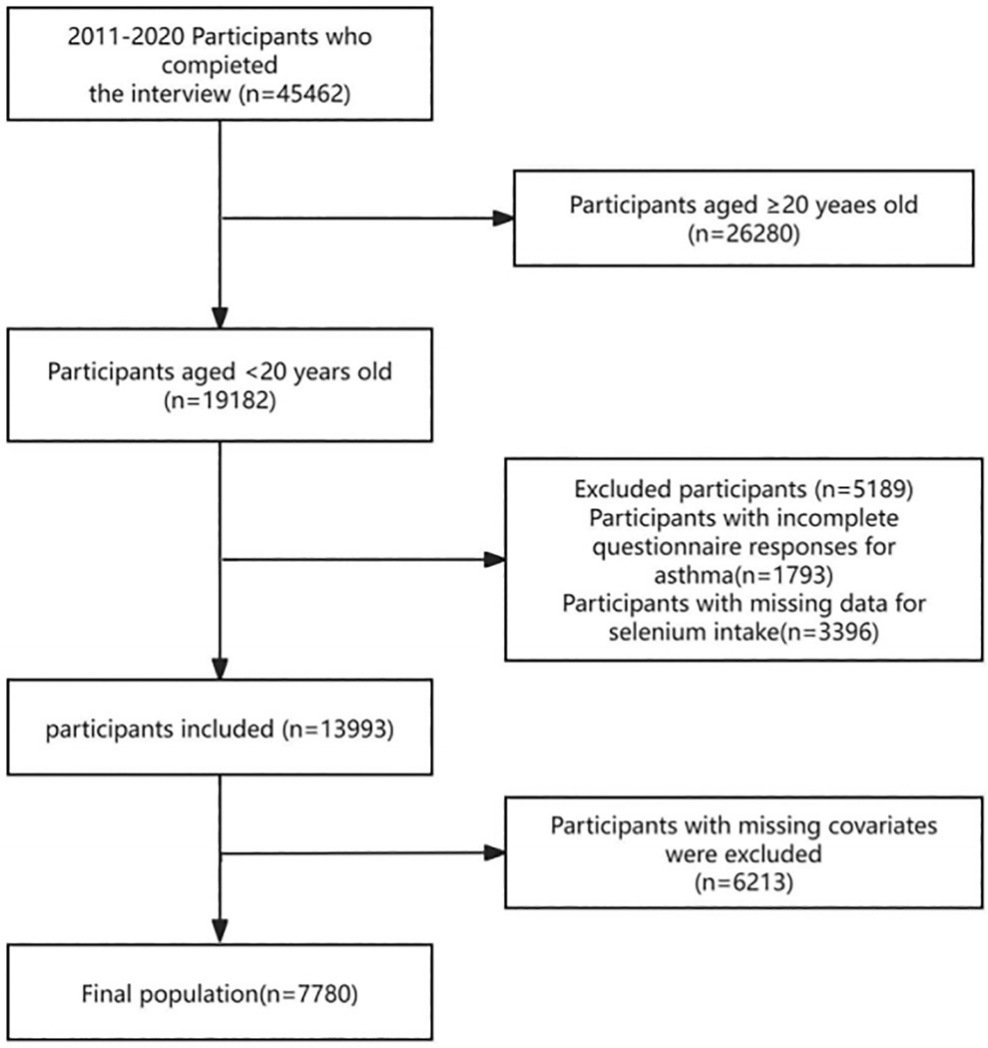
Illustration of the thorough screening procedure.

Conducted by the CDC [2], NHANES was designed to assess the health and nutrition status of the non-institutionalized US population through an annual survey of approximately 5000 individuals, constituting a nationally representative sample. Participants are selected using a multistage, stratified probability sampling design [12]. Data collection encompasses demographic information and extensive health-related data gathered via household interviews, physical examinations, and laboratory analyses performed at a mobile examination center (MEC). Ethical approval for the NHANES protocol was granted by the Ethics Review Board of the National Center for Health Statistics (NCHS). Written informed consent was obtained from all participants prior to enrollment. Notably, for this secondary analysis of publicly available, de-identified data, no additional institutional review board approval was required. The NHANES datasets are publicly accessible via the NHANES website (http://www.cdc.gov/nchs/nhanes.htm). The participants without asthma were excluded. Additionally, individuals lacking complete dietary selenium intake data or asthma questionnaire responses were excluded. The study cohort comprised participants aged < 20 years who completed the interviews. Data sources included screening, dietary, laboratory, and questionnaire components (Figure 1).

### Asthma assessment

To ascertain asthma status, participants' responses to the medical condition questionnaire item “Has a healthcare provider ever diagnosed you with asthma?” were evaluated. Affirmative responses categorized individuals as asthmatic; negative responses defined the asthma-free cohort.

### Dietary selenium intake

NHANES dietary survey participants reported all food and beverage consumption during a 24-hour period. Dietary intake data were collected from 2011 to 2020 using the Automated Multiple Pass Method (AMPM). These data enabled precise nutrient value calculations based on consumed dietary items [13]. Participants were categorized into tertiles according to quantified dietary selenium intake.

### Covariates

Potential confounding covariates were identified through established evidence from prior epidemiological studies [14], including age, sex, race and ethnicity, family income, laboratory parameters (white blood cell count [WBC], eosinophils percent, hemoglobin, zinc, protein, carbohydrate, sugar, fiber, and fat, and familial asthma. Ethnic classifications comprised Mexican American, other Hispanic, non-Hispanic White, non-Hispanic Black, and other races. Family income was categorized using the poverty income ratio (PIR) as low (<1.3), medium (1.3–35), or high (>3.5) Hematological parameters including complete blood counts (CBC) and cellular morphology were quantified for all participants using the Beckman Coulter DxH 800 system at NHANES mobile examination centers, following standardized procedures, including white blood cell count, eosinophil percentage, and hemoglobin. A dietary recall interview was conducted before MEC interviews to collect participants’ nutritional data for a 24-hour period, including zinc, protein, carbohydrates, sugar, fiber, and fat consumption. The NHANES questionnaire evaluated whether participants had familial asthma based on responses to: “Were blood relatives, both living and deceased, advised by a health professional that they had asthma?” Family members were classified as asthmatic if responses were affirmative; otherwise, they were classified as asthma-free. All covariates were excluded following rigorous assessment of their statistical relevance and potential confounding effects. All operational definitions conform to NHANES demographic variable specifications outlined in the standardized documentation [15].

### Statistical analysis

The NHANES dataset underwent secondary analysis. Categorical variables were reported as percentages; continuous variables as mean ± standard deviation or median (interquartile range), contingent upon distribution normality. Dietary selenium intake was stratified into tertiles. Intergroup heterogeneity was assessed using ANOVA (normally distributed data), Kruskal-Wallis tests (non-parametric distributions), and chi-square tests (categorical comparisons). Dietary selenium intake served as the primary exposure variable, with all analyses stratified by intake tertile. Logistic regression models generated odds ratios (ORs) with 95% confidence intervals (CIs) quantifying selenium-asthma associations. Model 1 adjusted for sociodemographic covariates (age, sex, race, ethnicity, PIR). Model 2 additionally incorporated clinical parameters: familial asthma history, eosinophil percentage, white blood cell counts, and hemoglobin levels. Model 3 further included nutritional covariates (zinc, protein, carbohydrate, sugar, fiber, and fat intake). Restricted cubic splines (RCS) with three knots assessed potential non-linear dose-response relationships between selenium intake and asthma prevalence following full covariate adjustment per Model 3. Stratified analyses across demographic strata (gender, age, PIR categories, ethnicity classifications, and asthma family history) further examined selenium-asthma associations. All statistical procedures were implemented using R software (version 4.2.2; R Foundation) with Free Statistics 2.1.1 platform integration. Statistical significance was defined as two-sided *p* < 0.05.

## Results

### Baseline characteristics of participants

The analytical cohort comprised 7780 U.S. children and adolescents stratified into selenium intake tertiles: Q1 (n = 2592), Q2 (n = 2591), and Q3 (n = 2597). Significant demographic variations were observed: males predominated in Q3 (63.4% vs 40.9% in Q1, *p* < 0.001), and participants in Q3 were older (mean 13.0 ± 3.8 years vs 12.0 ± 4.0 in Q1-Q2, *p* < 0.01). Ethnic distribution differed significantly (*p* = 0.011), with higher proportions of non-Hispanic blacks in Q1 (26.9% vs 23.8% in Q3). Nutritionally, all macro/micronutrient intakes exhibited dose-dependent increases across tertiles (all *p* < 0.001), with zinc intake rising from 6.3 ± 3.6 mg/day (Q1) to 14.3 ± 7.2 mg/day (Q3). Hematological parameters showed no significant intergroup differences except hemoglobin (13.7 ± 1.3 g/dL in Q3 vs 13.3 ± 1.2 in Q1, *p* < 0.001). Familial asthma history was comparable across tertiles (*p* = 0.363) (Table 1).

**Table 1:** Baseline characteristics of the study participants.

		Selenium intake (mg/day)			
Variables	Total (n = 7780)	Q1 (n = 2592)	Q2 (n = 2591)	Q3 (n = 2597)	*p*
Sex, n (%)					< 0.001
Male	3975 (51.1)	1060 (40.9)	1269 (49)	1646 (63.4)	
Female	3805 (48.9)	1532 (59.1)	1322 (51)	951 (36.6)	
Age, Mean ± SD	12.3 ± 3.9	12.0 ± 3.9	12.0 ± 4.0	13.0 ± 3.8	< 0.001
Race and ethnicity, n (%)					0.011
Mexican American	1564 (20.1)	496 (19.1)	538 (20.8)	530 (20.4)	
Other Hispanic	813 (10.4)	272 (10.5)	286 (11)	255 (9.8)	
Non-Hispanic White	2223 (28.6)	748 (28.9)	738 (28.5)	737 (28.4)	
Non-Hispanic Black	1974 (25.4)	696 (26.9)	660 (25.5)	618 (23.8)	
Other/Multiracial	1206 (15.5)	380 (14.7)	369 (14.2)	457 (17.6)	
PIR, n (%)					0.009
Low	3425 (44.0)	1196 (46.1)	1118 (43.1)	1111 (42.8)	
Medium	2769 (35.6)	907 (35)	955 (36.9)	907 (34.9)	
High	1586 (20.4)	489 (18.9)	518 (20)	579 (22.3)	
WBC (1000 cells/μL)	7.0 ± 2.1	7.0 ± 2.1	7.0 ± 2.1	7.0 ± 2.1	0.495
Eosinophils percent, n(%)	2.5 (1.6, 4.4)	2.5 (1.5, 4.3)	2.5 (1.6, 4.4)	2.6 (1.6, 4.4)	0.346
Hemoglobin(g/dL)	13.5 ± 1.3	13.3 ± 1.2	13.4 ± 1.2	13.7 ± 1.3	< 0.001
Zinc(mg/day)	10.1 ± 6.2	6.3 ± 3.6	9.6 ± 4.4	14.3 ± 7.2	< 0.001
Protein(mg/day)	71.7 ± 38.7	40.7 ± 15.7	66.4 ± 15.2	108.0 ± 41.5	< 0.001
Carbohydrate(mg/day)	259.3 ± 116.6	192.2 ± 80.9	253.2 ± 88.6	332.2 ± 128.5	< 0.001
Sugar(mg/day)	116.7 ± 67.7	94.4 ± 54.1	115.9 ± 59.7	139.8 ± 78.8	< 0.001
Fiber(mg/day)	14.5 ± 8.3	10.5 ± 6.4	14.1 ± 6.6	18.9 ± 9.3	< 0.001
Fat(mg/day)	78.3 ± 42.3	50.9 ± 24.7	74.8 ± 28.6	109.2 ± 47.1	< 0.001
Familial asthma, n (%)					0.363
No	5081 (65.3)	1665 (64.2)	1710 (66)	1706 (65.7)	
Yes	2699 (34.7)	927 (35.8)	881 (34)	891 (34.3)	

Abbreviations: age, sex, race and ethnicity, PIR, family asthma, eosinophils percent, WBC, hemoglobin, zinc, protein, carbohydrate, sugar, fiber, fat.

### Association between dietary selenium intake and asthma

Multiple logistic regression analyses quantified a statistically significant positive association between dietary selenium intake and pediatric asthma risk following full covariate adjustment (*p* = 0.042). Compared with the reference tertile (Q1: ≤ 73.3 mg/day), elevated asthma risk was observed in Q2 (73.4–112.6 mg/day; OR = 1.15, 95% CI: 0.98–1.35) and Q3 (≥ 112.7 mg/day; OR = 1.24, 95% CI: 1.00–1.54). This biologically plausible dose-response relationship suggests dietary selenium may modulate immunological pathways in childhood asthma development, warranting further mechanistic investigation (Table 2).

**Table 2:** Linear regression model analysis elucidates the correlation between dietary selenium intake and asthma.

Quartile	OR(95% CI)									
	No.	n(%)	crude	*P*-value	Model 1	*P*-value	Model 2	*P*-value	Model 3	*P*-value
Selenium intake (mg/day)										
Q1(≤73.3)	2592	464 (17.9)	1(Ref)		1(Ref)		1(Ref)		1(Ref)	
Q2(73.4∼112.6)	2591	510 (19.7)	1.12 (0.98∼1.29)	0.101	1.12 (0.98∼1.29)	0.104	1.16 (1∼1.34)	0.056	1.15 (0.98∼1.35)	0.082
Q3(≥112.7)	2597	561 (21.6)	1.26 (1.1∼1.45)	0.001	1.21 (1.05∼1.4)	0.007	1.24 (1.07∼1.44)	0.005	1.24 (1∼1.54)	0.048
Trend. Test	7780	1535 (19.7)		0.001		0.007		0.005		0.042

Note:.

Model 1: adjusted for age, sex, race and ethnicity, PIR.

Model 2: adjusted for Model1+family asthma, eosinophils percent, WBC, hemoglobin.

Model 3: adjusted for Model2+zinc, protein, carbohydrate, sugar, fiber, fat.

### Dose-response relationship between dietary selenium intake and asthma

**Fig. 2:**
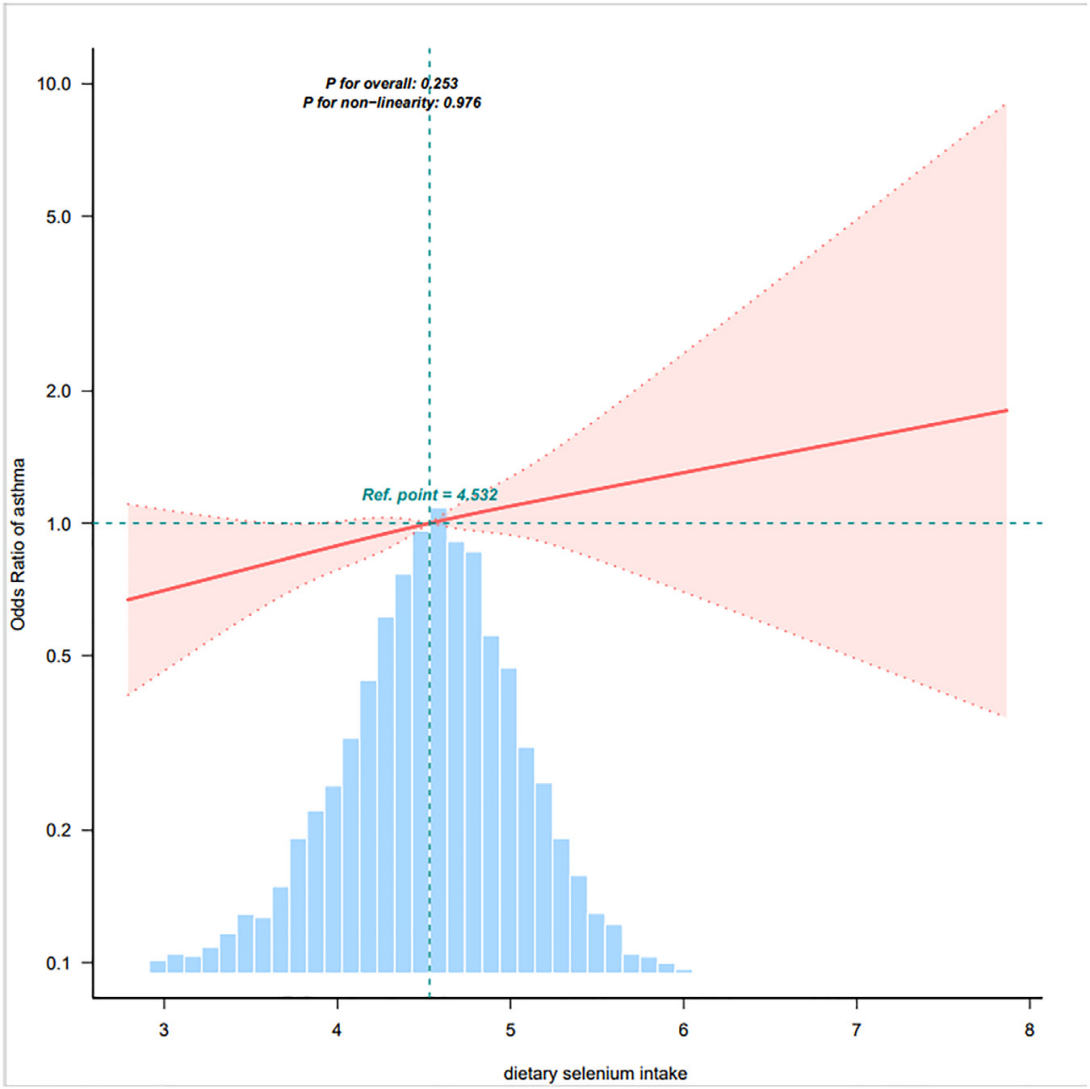
Association between dietary selenium intake and asthma odds ratio. They were adjusted for age, sex, race and ethnicity, PIR, family asthma, eosinophils percent, WBC, hemoglobin, zinc, protein, carbohydrate, sugar, fiber, fat. Only 99% of the data is shown.

Restricted cubic spline (RCS) analysis demonstrated a non-significant linear association between total dietary selenium intake and pediatric asthma risk (*p* = 0.976), following truncation of the lowest 0.1 percentile intake values and comprehensive adjustment for Model 3 covariates. The derived dose-response curve further revealed a statistically significant increasing trend in asthma prevalence with elevated selenium intake (β = 1.18, 95% CI: 1.05–1.32; *p* = 0.021) (Figure 2).

### Stratified analysis based on covariates

**Fig. 3:**
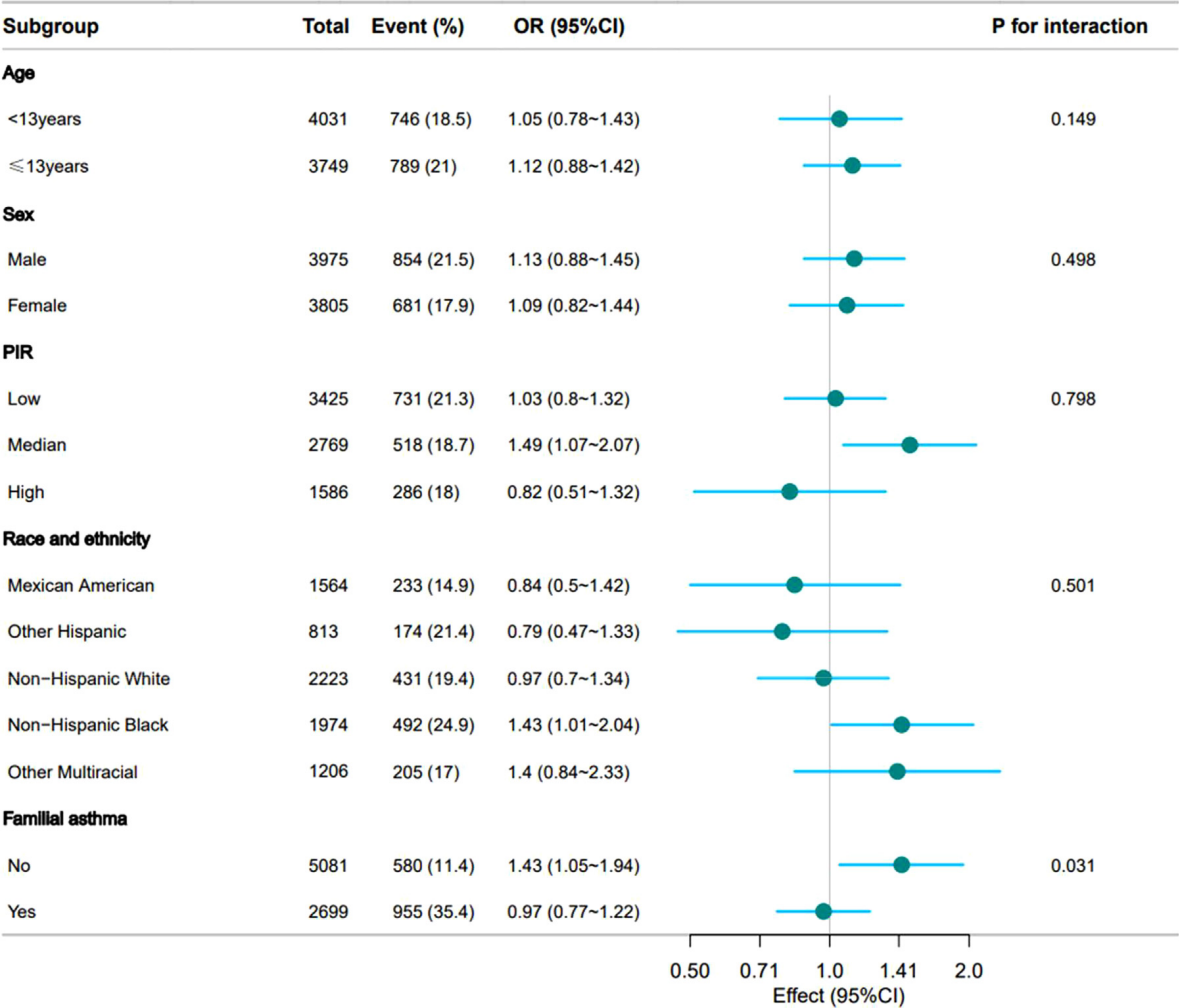
Forest plot of multivariate logistic analysis between dietary selenium intake and asthma. Except for the stratification component itself, each stratification factor was adjusted for age, sex, race and ethnicity, PIR, family asthma, eosinophils percent, WBC, hemoglobin, zinc, protein, carbohydrate, sugar, fiber, fat.

Stratified analyses across predefined subgroups confirmed the robustness of the primary selenium-asthma association, statistical interaction was quantified through likelihood ratio tests comparing models with and without cross-product terms, with persistently positive correlations between dietary selenium intake and asthma-related eosinophil counts evidenced in all demographic strata (*p* > 0.05). However, significant effect modification by familial asthma predisposition was observed (*p* = 0.031). These findings warrant validation in independent cohorts to elucidate causal relationships (Figure 3).

## Discussion

The large-scale analysis of nationally representative data demonstrates a significant positive association between dietary selenium intake and asthma prevalence among U.S. children and adolescents. This finding aligns with emerging evidence suggesting complex dose-response relationships between micronutrients and respiratory outcomes [8]. The observed adjusted OR of 1.24 (95% CI: 1.00–54) in the highest intake tertile (≥112.7 mg/day) suggests a clinically relevant 24% increase in asthma risk compared to the lowest intake group, challenging conventional assumptions about selenium's uniformly protective role [9].

These results provide crucial context for previous contradictory findings. The present data support the U-shaped relationship hypothesis wherein both deficiency and excess may be detrimental. Notably, the highest tertile exceeds current dietary reference intakes (DRIs) of 45–60 mg/day for children [16], potentially explaining why earlier studies with lower exposure ranges failed to detect such associations [17]. Selenium exerts dual immunomodulatory effects on immune function, though the precise mechanisms underlying its pro-allergic activity remain elusive. Substantial evidence implicates oxidative stress as the principal pathogenic mediator in allergic conditions, including asthma, allergic rhinitis, and atopic eczema. Mechanistically, excessive selenium may disrupt redox homeostasis through selenoprotein overexpression. Selenium (Se) serves as an essential cofactor for antioxidant enzymes that scavenge reactive oxygen species (ROS) [18]. Additionally, Se promotes Th1-mediated antiviral immunity, thereby suppressing inflammatory pathways [19]. Experimental evidence indicates Se deficiency alters human airway epithelial morphology and impairs influenza response [20], while rodent models demonstrate elevated macrophage PGE2 and TGF-β production during Se deprivation [21]. Collectively, these observations indicate Se may confer asthma protection through ROS mitigation and inflammation control. Paradoxically, the present analysis revealed a dose-dependent increase in asthma risk with higher selenium intake. This counterintuitive association may reflect selenium's immunomodulatory effects, potentially enhancing allergic sensitivity through amplified helper T-cell responses and immunoglobulin production [8]. Notably, epidemiological studies report no asthma prevalence elevation in Se-deficient populations [22], suggesting isolated selenium exposure may not directly drive asthma pathogenesis. Given the present cohort's predominantly mild or stable asthma phenotypes. Selenium supplementation demonstrates paradoxical effects in pediatric asthma cohorts, necessitating reevaluation of its therapeutic utility for asthma exacerbations [[23], [24]]. Future investigations should examine potential threshold effects and severe asthma subphenotype associations.

### Strengths and limitations

The present study possesses notable methodological strengths, including the integration of multiple NHANES survey cycles to establish a nationally representative sample, thereby augmenting statistical robustness and generalizability. Additionally, the analytical framework employed multivariable logistic regression complemented by stratified analyses, permitting rigorous examination of selenium-asthma associations. Notably, certain limitations warrant consideration. The NHANES database lacks longitudinal asthma progression metrics, precluding assessment of selenium's temporal relationship with disease evolution. Furthermore, the cross-sectional design inherently precludes causal inference. Future large-scale prospective cohorts and multicenter randomized controlled trials should prioritize elucidating causal pathways between dietary selenium exposure and pediatric asthma outcomes.

## Conclusion

This analysis demonstrates a significant positive association between elevated dietary selenium intake and heightened asthma risk among U.S. pediatric populations under 20 years of age. Dietary selenium exposure emerges as a modifiable determinant influencing pediatric asthma development, demonstrating a dose-dependent risk association.

## Ethics approval

The NHANES protocol obtained approval from the National Center for Health Statistics (NCHS) Ethics Review Committee, with informed consent secured from all participants prior to enrolment. No further institutional review board approval was necessary for this secondary analysis. The procedures used in this study adhere to the tenets of the Declaration of Helsinki.

## Funding

This research did not receive any specific grants from funding agencies in the public, commercial, or not-for-profit sectors.

## Authors’ contributions

(1) The conception and design of the study. (Lingyu Li, Shuai Wang)
(2) Acquisition of data. Analysis and interpretation of data. (Lingyu Li, Hongyan Wei)
(3) Drafting the article or revising it critically for important intellectual content. Final approval of the version to be submitted. (Lingyu Li, Changcheng Sun)

All authors read and approved the final manuscript.

## Data availability

The datasets supporting this study are available from the corresponding author upon reasonable request.

## Conflicts of interest

The authors declare no conflicts of interest.
